# Zinc protects against shiga-toxigenic *Escherichia coli* by acting on host tissues as well as on bacteria

**DOI:** 10.1186/1471-2180-14-145

**Published:** 2014-06-05

**Authors:** John K Crane, Jackie E Broome, Ryan M Reddinger, Benjamin B Werth

**Affiliations:** 1 Department of Medicine, Division of Infectious Diseases, University at Buffalo, Room 317 Biomedical Research Bldg, 3435 Main St, Buffalo, NY 14214, USA; 2 Department of Microbiology and Immunology, University at Buffalo, Buffalo, NY, USA

**Keywords:** Enterohemorrhagic *E. coli*, O157:H7, Hemolytic-uremic syndrome, SOS response, Diarrheal diseases, Xanthine oxidase, Manganese, Copper

## Abstract

**Background:**

Zinc supplements can treat or prevent enteric infections and diarrheal disease. Many articles on zinc in bacteria, however, highlight the essential nature of this metal for bacterial growth and virulence, suggesting that zinc should make infections worse, not better. To address this paradox, we tested whether zinc might have protective effects on intestinal epithelium as well as on the pathogen.

**Results:**

Using polarized monolayers of T84 cells we found that zinc protected against damage induced by hydrogen peroxide, as measured by trans-epithelial electrical resistance. Zinc also reduced peroxide-induced translocation of Shiga toxin (Stx) across T84 monolayers from the apical to basolateral side. Zinc was superior to other divalent metals to (iron, manganese, and nickel) in protecting against peroxide-induced epithelial damage, while copper also showed a protective effect.

The SOS bacterial stress response pathway is a powerful regulator of Stx production in STEC. We examined whether zinc’s known inhibitory effects on Stx might be mediated by blocking the SOS response. Zinc reduced expression of *recA*, a reliable marker of the SOS. Zinc was more potent and more efficacious than other metals tested in inhibiting *recA* expression induced by hydrogen peroxide, xanthine oxidase, or the antibiotic ciprofloxacin. The close correlation between zinc’s effects on *recA*/SOS and on Stx suggested that inhibition of the SOS response is one mechanism by which zinc protects against STEC infection.

**Conclusions:**

Zinc’s ability to protect against enteric bacterial pathogens may be the result of its combined effects on host tissues as well as inhibition of virulence in some pathogens. Research focused solely on the effects of zinc on pathogenic microbes may give an incomplete picture by failing to account for protective effects of zinc on host epithelia.

## Background

Zinc has been tested for its ability to treat and prevent diarrheal diseases in many large field trials over a period of over 4 decades [1-3] and has generally been found effective. Nevertheless, the protective mechanism of zinc has remained elusive. For example, most of the articles on zinc and enteric pathogens emphasize the essential nature of this metal and imply that zinc would enhance enhance the virulence of the pathogen [4,5] rather than help the host. It is often suggested that zinc acts via the immune system [6], but actual studies on zinc and immune responses are more nuanced and show that zinc can impair as well as enhance immune functions [7-10]. Instead of invoking zinc effects on immunity, we and others have shown that zinc can have pathogen-specific protective effects by acting directly on enteric bacteria including enteropathogenic *E. coli* (EPEC), Shiga-toxigenic *E. coli* (STEC), and enteroaggregative *E. coli* (EAEC) [11-13]. Recently, Mukhopadhyay and Linstedt reported that manganese could block the intracellular trafficking of Shiga toxin 1 (Stx1) and thus inhibit its ability to kill susceptible host cells [14]. This prompted us to reexamine the effects of zinc on host cells and to compare the effects of zinc with that of other divalent metals, including manganese.

STEC includes older names and subsets including enterohemorrhagic *E. coli*, EHEC, and Verotoxigenic *E. coli*, VTEC. STEC is the main cause of episodic “*E. coli* outbreaks” which are usually food-borne and often attract a great deal of attention in the news media [15-17]. As the name implies, these strains produce potent cytotoxins such as Stx1 or Stx2, or both. Absorption of Stx from the gastrointestinal tract can lead to severe extra-intestinal effects, including kidney failure, brain damage, and death. Antibiotics often make STEC infections worse by virtue of their ability to induce Stx production [18,19] and so are considered contraindicated in STEC infection. The severe sequelae of STEC infection has prompted many to seek additional treatments, sometimes by heroic measures that might rescue patients from the throes of full-blown disease, such as hemolytic-uremic syndrome (HUS) [20,21]. In contrast, we thought it would make more sense to intervene earlier in the course of STEC infection and prevent STEC infections from progressing to severe disease. Safe and inexpensive measures such as supplementation with oral zinc or other metals therefore seemed attractive as options. In contrast to our previous studies emphasizing the effects of zinc and other metals on the pathogenic bacteria, in this study we began by comparing zinc and other metals for protective effects on host epithelial cells, using T84 colonic cells grown as polarized monolayers. We found that zinc increased the trans-epithelial electrical resistance (TER) of the T84 cell monolayers; TER serves as a measure of epithelial integrity and of the barrier function provided by tight junctions. Zinc also protected monolayers from damage induced by hydrogen peroxide, an oxidant host defense that is released in response to EPEC and STEC infection [22,23]. We also examined if zinc and other metals had any effect of the translocation of Stx across T84 monolayers and found that it reduced toxin translocation as well. We also reexamined the ability of zinc to inhibit Stx production from STEC bacteria and correlated it with zinc’s ability to block the onset of the SOS bacterial stress response, as measured by *recA* expression, an early and quantifiable marker of the SOS response. While other metals occasionally mimicked zinc’s effects in one particular attribute or another, zinc was unique in its ability to simultaneously exert protective effects on host tissues while also inhibiting multiple bacterial pathways associated with STEC virulence such as the *recA*/SOS response, EHEC secreted proteins (Esps), the adhesins intimin and Tir, and Stx production. No other metal tested showed the same broad combination of beneficial effects as did zinc.

## Methods

### Bacterial strains used

Bacterial strains used are listed in Table 1. Bacteria were grown overnight in LB broth at 37°C with 300 rpm shaking, then subcultured into the medium for the expression studies, usually DMEM medium or minimal medium. In this report, when bacteria were subcultured in “DMEM” this refers to DMEM/F12 medium supplemented with 18 mM NaHCO_3_ and 25 mM HEPES, pH 7.4, but without serum or antibiotics.

**Table 1:** Bacterial strains used

**Strain name**	**Pathotype/serotype**	**Comment**	**Reference**
Popeye-1	STEC; O157:H7	*stx2; stx2c* United States 2006 spinach-associated outbreak strain.	[12]
EDL933	STEC; O157:H7	*stx1; stx2*	[23]
TSA14	STEC O126:H11	*stx1*	[23]
JLM281	*recA-lacZ* reporter strain derived from laboratory strain MC4100	*recA* is used as a measure of the SOS response to DNA damage in *E. coli*	[24]
JLM165	*LEE4-lacZ* reporter strain	LEE4 encodes the EPEC and EHEC secreted proteins (Esps)	[25]
KMTIR3	*LEE5-lacZ* reporter	LEE5 encodes Tir and intimin	[26]
mCAMP	*bla-lacZ reporter*	β-lactamase	[25]
MG1655	Used as susceptible host strain for bacteriophage plaque assays.		[27]

### Assays using T84 cells grown in polarized monolayers in Transwell inserts

T84 cells were grown to confluency over 7 to 10 days on 12 mm Transwell inserts (Corning Life Sciences, Lowell, MA) in T84 medium with 8% fetal bovine serum and antibiotics as described. The Transwells were of 0.4 μm pore size polycarbonate plastic, and were not coated with collagen or other proteins. Trans-epithelial electrical resistance (TER) was measured using an Evom2 meter (World Precision Instruments, Tampa, FL) and the STX2 chopstick electrode. (It is mere coincidence that the electrode has a name similar to the toxin we were studying.) We adjusted the concentration of hydrogen peroxide used to damage the monolayers based on the TER at the start of the experiment: 2 mM H_2_O_2_ was used for monolayers with resistances of 1000–1500 Ω, and 3 mM H_2_O_2_ for monolayers with resistances above 1500 Ω. TER values are reported in ohms (Ω). To obtain values in Ω · cm^2^, one would multiply by the area (1.12 cm^2^). For monolayer experiments, we removed serum-containing medium and performed the experiments in serum-free medium. Delta TER (ΔTER) is defined as the TER_final_ – TER_initial_; TER and Stx translocation measurements were done in quadruplicate wells and are shown as means ± SD.

### Stx toxin translocation assay

We measured translocation of Stx2 from the upper chamber to lower chamber in T84 cells grown in Transwell inserts (apical-to-basolateral) as described by Acheson et al. [28]. T84 cells are insensitive to the toxic effects of Stx, at least in part due to low or absent expression of the Gb_3_ glycolipid receptors for Stx1 and Stx2; intestinal epithelia in humans and other mammals also show nil expression of Gb_3_. As a source of Stx2 we used crude supernatants of STEC strain Popeye-1, subjected to sterile filtration, and containing 1 to 1.5 μg/mL of Stx2. Crude supernatant was used because other soluble factors present in STEC supernatants, including EHEC secreted protein P (EspP) increase the ability of Stx to translocate across monolayers by the trans-cellular route [29,30]. This crude supernatant would be expected to contain Stx2c as well as Stx2. Stx supernatants were diluted to a final concentration of Stx2 in the upper chamber of between 50,000 to 100,000 pg/mL in various experiments done over several months. Stx2 addition was delayed until 2 h after the oxidant in order to avoid denaturing the Stx by oxidation. Medium from the lower chambers was collected at various times and Stx2 measured by enzyme immunoassay (EIA) as described [12] using the Premier EHEC toxin EIA kit (Meridian Biosciences, Cincinnati, OH). Purified Shiga toxin 2 toxoid was a kind gift of Dr. Alison Weiss, Univ. of Cincinnati, and was used to create standard curves to allow better quantitation. To provide context, in monolayers damaged with 3 mM H_2_O_2_, the amount of Stx2 translocated across the monolayer at 24 h averaged 7.0 ± 4.8% of the amount originally added. Hypoxanthine + XO triggered a similar amount of Stx2 translocation: 8.5 ± 3.0% at 24 h (mean ± SD of 5 experiments).

### Miller assay for expression of β-galactosidase in bacterial reporter strains

Strain JLM281, the reporter strain containing the *recA*-*lacZ* construct was used to measure *recA* expression in response to inducing antibiotics, zinc and other metals. We used a version of the Miller assay adapted to 96 well plates for higher throughput [31]. However, we used 0.1% hexadecyltrimethylammonium bromide (HTA-Br) detergent alone, without chloroform or sodium dodecyl sulfate (SDS), to permeabilize the bacteria. The buffers used are described in a Open WetWare website at http://openwetware.org/wiki/Beta-Galactosidase_Assay_%28A_better_Miller%29.

Briefly, we subcultured strain JLM281 at a dilution of 1:100 from an overnight culture in DMEM into a 96 well plate containing minimal medium, 150 μl per well, on a Bioshake iQ thermal mixer (Quantifoil Instruments GmbH, Jena, Germany) at 37°C with mixing at 1200 rpm. We used DMEM for these expression experiments because induction of *recA*, LEE4, and LEE5 were higher in DMEM than in LB broth. The 96 well plate was sealed with gas-permeable plate sealing film to prevent evaporation during the growth phase. At 4 h when the cultures reached an OD_600_ in the 0.2 to 0.3 range, 20 μl of bacterial culture was transferred to the wells of a a second 96 well plate containing 80 μl of permeabilization buffer and allowed to permeabilize for at least 10 min at room temperature. The β-galactosidase reaction was initiated by transferring 25 μl of permeabilized bacteria into a third 96 well plate containing 150 μl of substrate solution with 1 g/L o-nitrophenyl-β-galactoside (ONPG). The enzyme reaction plate was incubated at 30°C for 30 min, and then A_420_ was measured on the 96 well plate reader. We usually omitted the addition of the Na_2_CO_3_ stop solution. Miller units were calculated using the simplified equation:

$$1000\times \frac{A_{420}}{OD_{600}\times \left(\mathrm{volumes}\;\mathrm{sampled}\;\left[0.02\mathrm{ml}\right]\right)\times \mathrm{reaction}\;\mathrm{time}\;\mathrm{in}\min\left[\mathrm{usually}30\right]}$$

### Agar overlay assay for bacteriophage plaques by modified spot assay

We used wild-type STEC strains as the source of bacteriophage for these experiments. STEC bacteria were subcultured at a dilution of 1:100 into antibiotic-free DMEM medium from an overnight culture. After 1 h of growth at 37°C with 300 rpm shaking, additions such as ciprofloxacin or zinc were made and the tubes returned to the shaker incubator for 5 h total. The STEC suspension was clarified by centrifugation, then subjected to sterile filtration using syringe-tip filters. The STEC filtrate was diluted 1:10 in DMEM medium, then serial 2-fold dilutions were made to yield dilutions of 1:20, 1: 40, 1: 80 and so on. The recipient strain, *E. coli* MG1655, was subcultured at 1: 50 from overnight and grown in LB broth for 3 hours. Soft LB agar was prepared using LB broth supplemented with 0.5% agar and 0.5 mM MgSO4. The soft agar was melted by microwave heating, and kept warm at 45°C on a heater block. The MG1655 culture was diluted 1: 10 into the soft agar and 5 ml of the bacteria-containing agar was overlaid on top of the agar of regular LB agar plate and allowed to solidify. Then 3 μl aliquots of the diluted STEC filtrates were spotted on top of the agar overlay. Plaques were visualized after 16 h of additional incubation at 37°C. Any faint zone of clearing was counted as a plaque. The highest dilution of STEC filtrate that produced a plaque was recorded as the plaque titer.

### Rabbit infection experiments

No new rabbit infection experiments were performed for this study. We used photographs from the archives of our previous animal experiments to create the illustration in final figure. Nevertheless, all of our past and ongoing animal work has been scrutinized and approved by the animal care committee (IACUC) of the University at Buffalo.

### Data analysis and statistics

Error bars shown on graphs and in Tables are standard deviations. Statistical signficance was tested by ANOVA using the Tukey-Kramer post-test for multiple comparisons.

## Results

We recently reported that the xanthine oxidase (XO) enzyme pathway is activated in response to EPEC and STEC infection [23]. Infection with these pathogens triggers a release of nucleotides and nucleosides into the gut lumen, and XO itself is also released into the lumen of the intestine as a result of damage inflicted by these pathogens. XO catalyzes the conversion of hypoxanthine to xanthine and xanthine to uric acid, with both steps creating one molecule of hydrogen peroxide. As previously reported by Wagner for oxidant molecules generated from neutrophils [22], XO-generated H_2_O_2_ increases the production of Stx from STEC strains [23]. Since H_2_O_2_ is known to be able to damage intestinal epithelia [32,33], we thought this would be a relevant model to test whether zinc or other metals could protect against oxidant damage, since zinc has been reported to reported to help restore intestinal barrier function following other insults [34]. We used T84 cells grown to confluency in polarized monolayers in Transwell inserts as previously reported [28]. We measured trans-epithelial electrical resistance (TER), an index of intestinal barrier function, as well as H_2_O_2_-induced translocation of Stx2 from apical to basolateral chambers.

**Figure 1:**
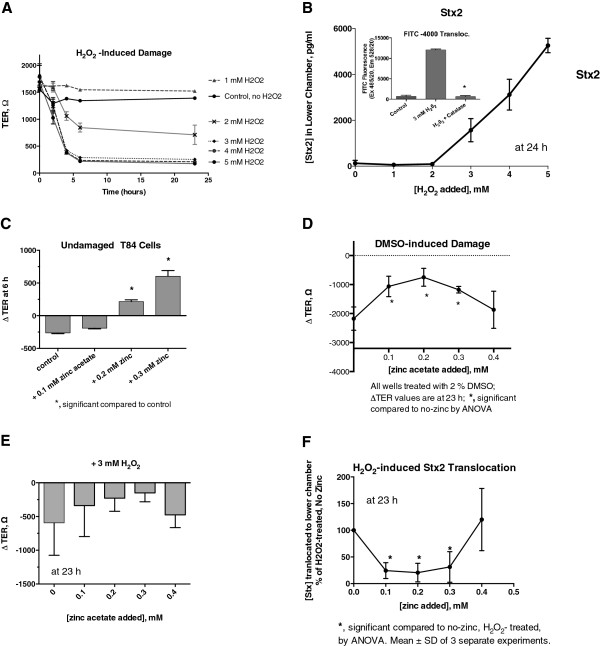
**Effect of zinc acetate on hydrogen peroxide-induced intestinal damage and Stx2 translocation in T84 cells.** T84 cells grown to confluency in Transwell inserts were treated with various concentrations of hydrogen peroxide and barrier function monitored by measuring trans-epithelial electrical resistance (TER) and translocation of Stx2 across the monolayers. Stx2 itself does not damage T84 cells due to lack of expression of the Gb_3_ receptor in this cell line. **Panel A**, time course of TER in response to H_2_O_2_ added to final concentrations of 1 to 5 mM. **Panel B**, effect of H_2_O_2_ on translocation of Stx2 and on fluorescein-labeled dextran-4000. Stx2 was added to the upper chamber 2 hours after the addition of H_2_O_2,_ and Stx2 was measured by EIA in the lower chamber. H_2_O_2_ at concentrations of 3 mM and higher induced significant translocation of Stx2 into the lower chamber. The amount of Stx2 translocated to the lower chamber after 24 in response to 5 mM H_2_O_2_ was 3.5% of the total Stx2 added. Panel **B**, *Inset*, shows that H_2_O_2_ also triggers a translocation of FITC-dextran-4000 across the monolayer, which is abolished by addition of 1200 U/mL of catalase; *significant compared to H_2_O_2_ alone. **Panels C**, effect of zinc acetate on Δ TER in undamaged T84 cell monolayers. Δ TER is defined as the TER_final_ – TER_initial_, which is determined separately for each well, then averaged. Using the Δ TER helps to compensate for well-to-well variation in the starting TER, because each well serves as its own control. **Panel D**, effect of zinc acetate on Δ TER in cells treated with 2% DMSO. **Panel E**, effect of zinc on T84 cell monolayers treated with 3 mM H_2_O_2_. **Panel F**, protection by zinc against Stx2 translocation induced by exposure to H_2_O_2_.

Figure 1 shows the effects of H_2_O_2_ on TER and Stx2 translocation. H_2_O_2_ damages tight junctions and increases permeability via the paracellular pathway [35]. Figure 1A shows that H_2_O_2_ has concentration-dependent and time-dependent effects on TER in the T84 monolayers. 1 mM H_2_O_2_ paradoxically increased TER slightly, but 2 mM H_2_O_2_ caused a moderate drop in TER. H_2_O_2_ at 3 mM and above damaged the monolayers severely, with TER falling to ~100 Ω, which is equivalent to that of the Transwell filters alone without any cells. Figure 1B shows that H_2_O_2_ also had a concentration dependent effect on Stx2 translocation, with Stx2 translocation detectable at H_2_O_2_ concentrations of 3 mM or higher. The inset in Figure 1B shows that H_2_O_2_ was also able to trigger a flux of fluorescein-labeled dextran-4000 across the monolayer, and that the monolayer damage could be prevented by the addition of catalase. Figure 1C shows that zinc could increase the TER in T84 cells not subjected to hydrogen peroxide or any other noxious stimulus, and Figure 1D shows that zinc could protect against the drop in TER induced by treatment with 2% dimethylsulfoxide (DMSO), at least at intermediate concentrations. Zinc acetate seemed to reduce the drop in TER (∆ TER) induced by 3 mM H_2_O_2_, although this protective effect did not reach statistical significance (Figure 1E). Figure 1F shows, however, that intermediate concentrations of zinc (0.1 to 0.3 mM) did significantly reduce Stx2 translocation across the T84 monolayers. At zinc concentrations of 0.4 mM and higher, however, the protective effect was lost, resulting in a U-shaped curve in Figure 1F (data not shown for concentrations greater than 0.4 mM). The U shape in Figure 1F seemed to mirror the arch shape of the curves in Figure 1D and E, and suggested that zinc might have interesting protective effects against insults to the intestinal epithelium.

**Figure 2:**
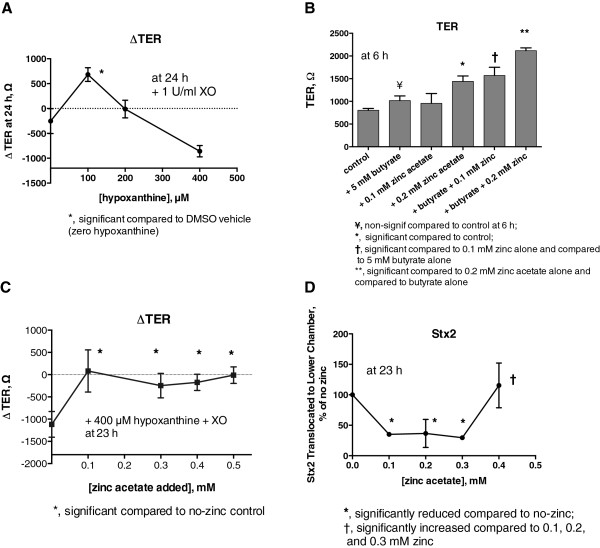
**Effect of hypoxanthine plus xanthine oxidase on barrier function and Stx2 translocation in T84 cells. Panels A-C** show effects on TER, while **Panel D** shows effect on Stx2 translocation. The “standard” concentration of hypoxanthine was 400 μM if not otherwise stated, and the standard concentration of XO was 1 U/mL. **Panel A**, effect of various concentrations of hypoxanthine on TER. The “zero” hypoxanthine condition received 1% DMSO vehicle alone. **Panel B**, additive effect of zinc with butyrate on TER. **Panel C**, protection by zinc against the drop in TER induced by hypoxanthine plus XO. **Panel D**, protection by zinc against Stx2 translocation triggered by hypoxanthine plus xanthine oxidase.

In Figure 1 the hydrogen peroxide was added once at fairly high concentrations, but in an actual infection the hydrogen peroxide (and other oxidants, such as superoxide and sodium hypochlorite) is generated gradually from enzymatic conversion of substrates over many hours. Therefore we repeated experiments similar to those shown in Figure 1, but instead using H_2_O_2_ we added hypoxanthine plus XO. Figure 2A shows that, in the presence of XO, hypoxanthine has a concentration-dependent effect on ∆ TER. Adding 100 μM hypoxanthine actually increased TER compared to vehicle control, with higher concentrations of hypoxanthine inducing a progressive fall in TER. The increase in TER observed in Figure 2A at 100 μM hypoxanthine was reminiscent of the small increase in TER seen with 1 mM H_2_O_2_ in Figure 1A (top curve). Figure 2, Panels B, shows that the effect of zinc on T84 cell monolayers was additive with the known protective effects of 5 mM sodium butyrate on colon cell monolayers [36]. The concentration of butyrate we used is well within the concentrations known to occur in the lumen of the lower gastrointestinal tract [37]. Figure 2C shows that zinc at 0.1 to 0.5 mM significantly protected cells from the drop in TER inflicted by XO + 400 μM hypoxanthine. Likewise, Figure 2D shows that 0.1 to 0.3 mM zinc, but not 0.4 mM zinc, reduced Stx2 translocation triggered by XO + 400 µM hypoxanthine. Thus, while Figure 2C did not show the arch shape seen in Figure 1C, Figure 2D does have the “U” shape similar to that seen in Figure 1D with hydrogen peroxide as the injuring oxidant. In monolayers treated with hypoxanthine + XO, the amount of Stx2 that translocated across the monolayer in 24 h was 8.5 ± 3.0% (mean ± SD of 5 experiments) of the total amount added to the upper chamber. Figures 1 and 2 showed that zinc acetate could protect against oxidant-induced drop in TER, a measure of intestinal barrier function, and inhibit the translocation of Stx2 across T84 cell monolayers as well.

**Figure 3:**
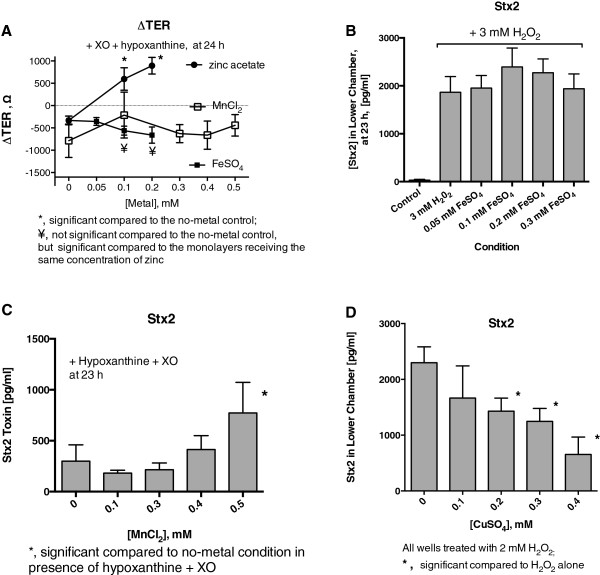
**Effect of metals other than zinc on oxidant-induced changes in TER and on Stx2 translocation.** As in Figure 2, the “standard” concentration of hypoxanthine was 400 μM if not otherwise stated and the “standard” amount of XO was 1 U/mL. **Panel A**, lack of protection by FeSO_4_ and MnCl_2_ on oxidant-induced ∆ TER. **Panel B**, lack of protection by FeSO_4_ on oxidant-induced Stx2 translocation. **Panel C**, lack of protection by MnCl_2_ on oxidant-induced Stx2 translocation. **Panel D**, protection by CuSO_4_ against oxidant-induced Stx2 movement across the monolayer.

In Figure 3 we examined the effects of other metals on TER and Stx2 translocation. We focused on the transition metals nearest to zinc in atomic number, including manganese, iron, nickel, and copper. Figure 3A shows the effects of two of these metals on TER, while Panels B-D show the effects on Stx2 translocation. Figure 3A shows that in contrast to zinc (top curve), FeSO_4_ and MnCl_2_ had no protective effect against the drop in TER triggered by XO + hypoxanthine. Copper (as CuSO_4_) also failed to protect against the drop in TER (data not shown). When Stx2 translocation was measured, FeSO_4_ seemed to slightly enhance Stx2 translocation triggered by H_2_O_2_ (Figure 3B), but this did not reach statistical significance. Nevertheless, iron has been shown to be able to potentiate oxidant-induced damage, and this has often been attributed to iron’s ability to catalyze the Fenton reaction, in which H_2_O_2_ is split into 2 molecules of hydroxyl radical (HO•). Figure 3C shows that manganese (as MnCl_2_) failed to protect against Stx22 translocation, and at 0.5 mM manganese significantly increased the amount of Stx2 crossing the monolayers. Notably, 0.5 mM was the effective concentration of manganese used by Mukhopadhyay and Linstedt [14] in their study of Stx1 trafficking in HeLa cells. Figure 3D shows that CuSO_4_, like zinc, significantly reduced Stx2 translocation. This was a surprise because of the lack of protection by CuSO_4_ on TER. Nickel chloride also had no protective effect on TER and none on Stx2 translocation at 0.1 to 0.5 mM (data not shown).

To summarize Figures 1, 2 and 3, zinc increased the TER in undamaged cells, and protected intestinal monolayers against the drop in TER induced by DMSO, by hydrogen peroxide, and that induced by XO plus hypoxanthine. Zinc also protected against oxidant-induced translocation of Stx2 across the monolayers at 0.1 to 0.3 mM concentration. These protective effects of zinc are attributable to actions of zinc on the host tissues, not on bacteria. None of the four other metals tested (iron, manganese, copper, or nickel) protected against oxidant-induced decrease in TER, but copper was still able to reduce Stx2 translocation across monolayers (Figure 3D). Our results did not support the idea, advanced by Mukhopadhyay and Linstedt, that manganese was the metal with the greatest promise for protection against STEC infection in the clinical setting [14]. Zinc still seemed to be a candidate for such studies, but to address this more fully we compared zinc and other metals for their ability to block bacterial signaling and stress-response pathways associated with virulence.

Stx production and release in STEC bacteria is strongly regulated by the SOS stress response system in *E. coli*[18,38]. In contrast, Stx production is quite insensitive to commonly mentioned signaling pathways such as quorum sensing, and to transcription factors such as the LEE-encoded regulator (Ler) and Plasmid-encoded regulator (Per) [25,39-41]. This is not surprising since *stx1* and *stx2* are encoded on phages similar to phage lambda, and these phage genes are strongly activated by the DNA damage triggered by certain antibiotics [18], hydrogen peroxide [22,42], or ultraviolet light. An early, reliable, and quantifiable marker of the SOS response is the expression of *recA*[43,44]. We hypothesized that zinc’s ability to inhibit Stx production arises from its ability to inhibit the SOS response and *recA*. To test this, we measured *recA* expression using a *recA-lacZ* reporter gene construct using the Miller assay method and compared those results with metals ability to inhibit Stx production.

**Figure 4:**
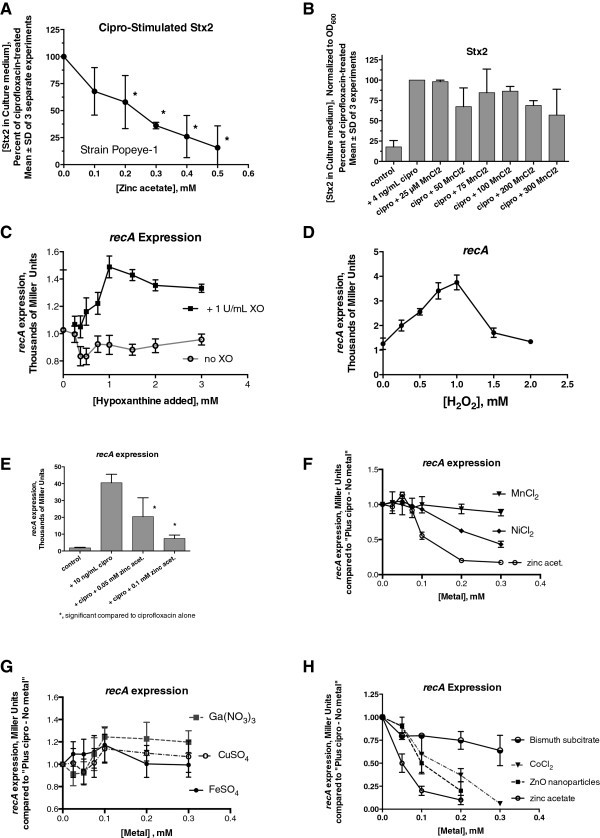
**Effects of zinc and other metals on Stx production from STEC, and on *recA *expression. Panels A** and **B**, effect of metals on production of Stx2 from STEC strain Popeye-1. In both panels, the results of 3 separate experiments are combined and expressed as a percent compared to the amount of Stx2 in the presence of 4 ng/mL ciprofloxacin alone (mean ± SD). *significantly reduced compared to the no-zinc control, by ANOVA. **Panels C-H**, expression of *recA* as measured in the Miller assay using reporter strain JLM281 (*recA*-lacZ). **Panel C**, effect of hypoxanthine ± XO on *recA* expression. Despite the lack of asterisks, *recA* expression was significantly higher in the presence of XO than in its absence for concentrations of hypoxanthine of 0.8 mM or higher. **Panel D**, H_2_O_2_ induction of *recA* expression in JLM281. **Panel E**, reversal of ciprofloxacin-induced *recA* expression by zinc. **Panels F-H**, comparison of other metals on *recA* expression, with results normalized as a ratio to that of the “plus ciprofloxacin, no metal” condition for each metal and concentration.

Figure 4A shows that zinc inhibits ciprofloxacin-induced Stx2 production strongly and in a dose-dependent manner. In contrast, MnCl_2_ had no such ability to inhibit either ciprofloxacin-induced Stx2 production (Figure 4B) or basal (non-antibiotic treated) Stx release [12]. Figure 4C shows that *recA* expression increased in reporter strain JLM281 when hypoxanthine is added in the presence of the enzyme XO, but not in the absence of XO. Hydrogen peroxide itself showed a *recA* activation curve with a similar shape (Figure 4D). Zinc acetate inhibited ciprofloxacin-induced *recA* expression (Figure 4E) as well as hydrogen-peroxide induced *recA* expression (data not shown). Zinc acetate was more efficacious and more potent in inhibition of ciprofloxacin-induced *recA* expression that MnCl_2_ or NiCl_2_ (Figure 4F) and more than FeSO_4_, CuSO_4_, or gallium nitrate (Figure 4G). Gallium was tested because of its position next to zinc on the Periodic Table and because others had reported it had anti-virulence activity [45]. Figure 4H shows that zinc acetate was more potent than zinc oxide nanoparticles, CoCl_2_, or bismuth subcitrate in inhibition of *recA* induced by ciprofloxacin. Bismuth was tested because of its long use as a treatment for infectious diarrhea [46,47], and zinc oxide nanoparticles were reported to have activity against *Campylobacter jejuni*[48]. In summary, zinc acetate was more potent and more effective in inhibiting ciprofloxacin-induced *recA* than any other metal shown in Figure 4. Zinc also blocked *recA* induced by mitomycin C (data not shown). As controls, zinc did not block the induction of other genes, including a β-lactamase-lacZ reporter gene (see final figure below), or the ability of isopropyl-thio-galactose (IPTG) to induce beta-galactosidase in wild-type *E. coli* strains (data not shown). We did not test metals such as cadmium, mercury, or lead, because we are interested in the translational use of these findings and felt those metals were too toxic to be considered for use in humans or animals.

**Figure 5:**
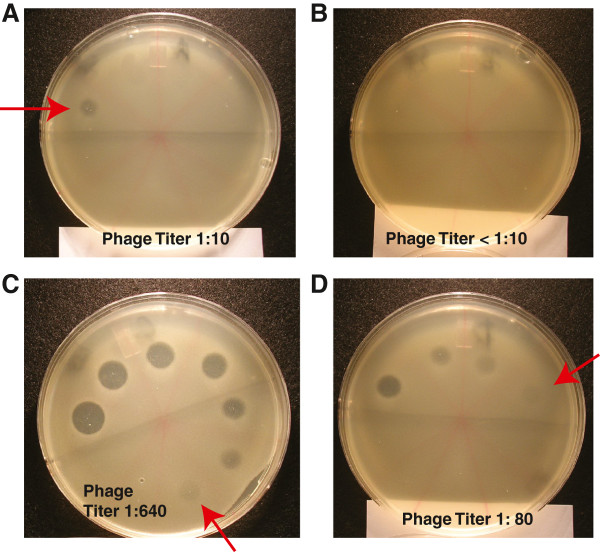
**Effect of zinc on ciprofloxacin-induced bacteriophage production from STEC bacteria, as assessed by a semi-quantitative “spot” assay.** STEC filtrates were prepared as described in Materials and Methods from strain TSA14 and diluted to 1:10, 1:20, 1:40, 1: 80, and so on to 1:2560. **Panel A**, sterile filtrate of TSA14 not treated with antibiotics or zinc, showing a phage titer of 1: 10. **Panel B**, STEC filtrate from bacteria treated with 0.4 mM zinc; no phage plaques are visible. **Panel C**, spot assay from TSA14 treated with 4 ng/mL ciprofloxacin, showing a titer of 1:640. **Panel D**, phage titer resulting from bacteria treated with ciprofloxacin and zinc, showing a 8-fold reduction in phage plaque titer compared to ciprofloxacin alone.

Since our finding that zinc-mediated inhibition of *recA* expression had not been previously reported, we tested whether zinc was actually blocking the entire bacterial SOS response, or merely preventing *recA* expression in an artefactual way. A reliable “downstream” marker of the SOS stress response in *E. coli* is a marked elongation of the bacterial cells, sometimes called filamentation, which is due to inhibition of the fission ring formed by FtsZ. We tested whether zinc inhibited antibiotic-induced elongation of bacteria. Additional file 1: Figure S1 shows that zinc reversed ciprofloxacin-induced bacterial elongation in EPEC E2348/69 and in STEC strain Popeye-1, as well as mitomycin C-induced elongation in Popeye-1. In contrast to zinc, manganese and nickel did not have any effect on antibiotic-induced elongation (Additional file 1: Figure S1B and 1C). Zinc also blocked the production of infectious bacteriophage from STEC strains Popeye-1, EDL933, and TSA14, as assessed by phage plaque assays on laboratory *E. coli* strain MG1655 (Figure 5 and Table 2). Therefore we conclude that zinc blocks all the core features of the SOS response, and not merely *recA* induction.

**Table 2:** **Effect of zinc on the bacteriophage yield from STEC bacteria by phage plaque assay on *E. coli *** **MG1655 as host strain**

**Experiment number**	**Donor/source strain for bacteriophage**	**Growth condition (in DMEM Medium)**	**Bacterio-phage titer**	**Fold reduction by zinc**
Expt. 1	**TSA14**; O26:H11, Stx1+; harbors phage H19B	control, no additives	1:10	
		+ 0.4 mM Zn	no plaques, < 1:10	> 2-fold decrease
		+ 4 ng/ml cipro	1:640	
		+ 4 cipro + 0.4 mM Zn	1:80	8-fold decrease
Expt. 2	**TSA14**; O26:H11	control, no additives	1:20	
		+ 0.6 mM Zn	no plaques	> 2-fold decrease
		+ 8 ng/ml cipro	1:640	
		+ 8 cipro + 0.4 mM Zn	1:160	4-fold decrease
		+ 8 cipro + 0.6 mM Zn	1:80	8-fold decrease
Expt. 3	**EDL933**; O157:H7; Stx1+, Stx2+;	control	1:80	
		+ 0.6 mM Zn	1:40	2-fold decrease
	Harbors phages H19B and 933 W	+ 10 ng/ml cipro	> 1:5120	
		+ 10 cipro + 0.6 mM Zn	1:320	≥ 16-fold decrease
Expt. 4	**EDL933**	control	1:80	
		+ 0.6 mM Zn	1:20	4-fold decrease
		+ 10 ng/ml cipro	1:640	
		+ 10 cipro + 0.6 mM Zn	1:160	4-fold decrease

All source strains were grown for 5 hours, 4 hours after addition of ciprofloxacin and/or zinc.

Zn, zinc acetate; cipro, ciprofloxacin, usually added at ~ 1/3 of the MIC.

**Figure 6:**
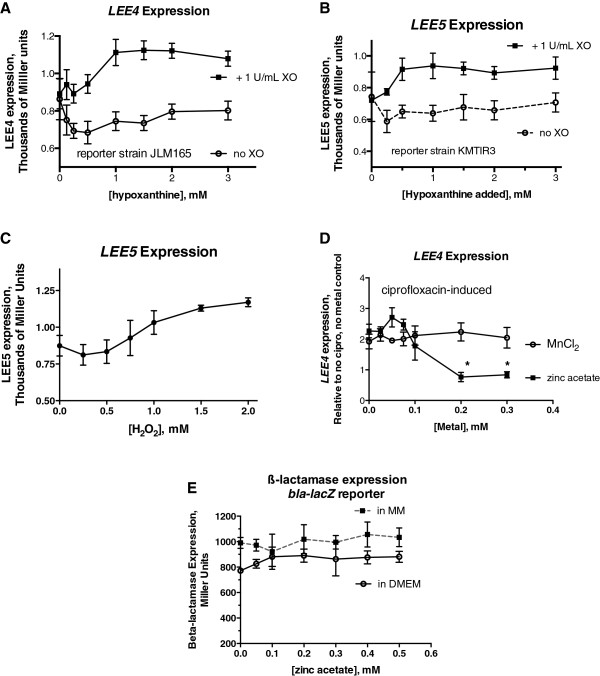
**Effect of zinc and other metals on expression of LEE operons as measured in reporter strains.** Reporter strains JLM165 (for LEE4, encoding the Esps) KMTIR3 (for LEE5, encoding Tir and intimin) and mCAMP (for beta-lactamase) were used to measure gene expression using the Miller assay. **Panels A** and **B**, expression of LEE4 and LEE5 were significantly increased in dose-dependent fashion by hypoxanthine in the presence of XO, compared to without added XO. **Panel C,** LEE5 expression was modestly but significantly increased in response to H2O2. **Panel D**, zinc acetate, but not MnCl_2_, inhibited induced LEE4 expression. *significant compared to “plus cipro, no-metal” condition. **Panel E**, lack of effect of zinc on expression of beta-lactamase in the *bla-lacZ* reporter strain in two different types of liquid media, minimal medium (MM) and DMEM.

Stx is an important virulence factor in STEC, but it is not the only one. Therefore, we also tested whether operons in the locus for enterocyte effacement (LEE) were activated by oxidant stress, and if so, whether, they were susceptible to inhibition by zinc. We used *LEE4-lacZ* and *LEE5-lacZ* reporter strains; LEE4 encodes the EPEC and EHEC secreted proteins (Esps), and LEE5 encodes the critical adhesins Tir and intimin, and the CesT chaperone. Figure 6 shows that, in the presence of XO, hypoxanthine substrate does modestly activate expression of both LEE4 (Figure 6A) and LEE5 (Figure 6B). Figure 6C shows that H_2_O_2_ also induced LEE5 expression in a manner similar to that triggered hypoxanthine plus XO, and as previously shown for ciprofloxacin [24]. Figure 6D shows that zinc acetate inhibited LEE4 expression, but unfortunately manganese chloride showed no such ability. Figure 6 shows first that LEE operons may be up-regulated by oxidant stress, and second that the virulence-inhibiting abilities of zinc extend to factors other than Stx including critical adhesins and Type III secreted proteins encoded in the LEE. While Figures 1, 2 and 3 focused on the protective effects of zinc and other metals on intestinal cells, Figures 4, 5 and 6 extend our previous understanding of zinc’s direct effects on bacteria [11,12], showing zinc’s ability to inhibit the SOS response as measured by *recA* expression (Figure 4), a property not matched by any other metal tested. The good correlation between zinc’s inhibition of *recA* expression (Figure 4), filamentation (Additional file 1: Figure S1), phage production, and zinc’s inhibition of Stx toxin protein (Figure 4A) and *stx* RNA [12] suggests that zinc’s ability to block *recA* activation is an important part of the mechanism of action of this metal in STEC and EPEC infection.

## Discussion and conclusions

Our understanding of the roles of divalent metals as regulators of bacterial pathogenesis has lagged behind that of other molecules such as quorum sensing auto-inducers and transcriptional regulators such as H-NS and Ler [49]. Most of the work on transporters and metabolism of zinc and other metals has been done with non-pathogenic laboratory strains of *E. coli*[50-52], which makes the results difficult to extrapolate to strains which are professional intestinal or extra-intestinal pathogens. For example, STEC expresses several different metal uptake and zinc export genes not present in laboratory *E. coli* strains [4,5,53,54] so STEC’s response to bioactive metals often differs from non-pathogenic *E. coli*. In addition, the specialized Type III secretion system (and Type VI secretion system in EAEC) used to deliver effectors into host cells may serve as an “Achilles’ heel” in these pathotypes because the membrane secretion machinery causes them to become hypersusceptible to some stressful stimuli [55] such as the envelope stress response [27,56]. Furthermore, many of the reports on zinc in enteric bacteria only focus on the essential nature of this metal for the pathogen [4,57], without consideration of how zinc might also benefit the host. In addition, many reports do not distinguish between the growth-and-fitness promoting effects of zinc on pathogens at the low concentrations usually present (1 to 50 μM) versus the higher, stress-inducing concentrations of zinc that can occur during zinc supplementation (0.1 to 0.4 mM). In general, it appears that host cells are better able to survive--- and thrive--- in the presence of these higher zinc concentrations that are deleterious to *E.coli* and other enteric bacteria ( [58,59], and Figures 1, 2 and 3 of this study). Moreover, studies that have actually tested zinc for infection outcomes using cultured cell models or animal models have generally shown that zinc benefits the host more than the pathogen, resulting in a reduction in severity of disease [11,13,48,60]. Indeed, Botella et al. recently showed that zinc is mobilized in macrophages and concentrated in phagosomes as part of the host defense against *Mycobacterium tuberculosis*[61]. This is relevant to the gut because zinc is also concentrated in the secretory granules of Paneth cells [62,63], specialized cells in the intestinal crypts involved in antimicrobial defenses.

The discovery that zinc specifically inhibits virulence factor expression by some pathogens and not others has led us to emphasize that zinc’s effects may be pathogen-specific [64]. We may have to temper that emphasis, however, because Figures 1 and 2 of this study show zinc may strengthen the intestinal epithelial barrier against oxidant damage and this might extend zinc’s protection to organisms that are not specifically affected by zinc. Zinc may have mild protective effects against multiple diarrheal pathogens via its effects on enterocytes, and then also have additional protective activity against specific pathogens such as EPEC, STEC, EAEC, and Campylobacter.

Mukhopadhyay and Linstedt reported that manganese was able to block the intracellular trafficking of Stx1 through the Golgi apparatus of Stx-susceptible HeLa cells engineered to overexpress the glycolipid Gb_3_[14]; by doing so MnCl_2_ appeared to block the toxic effects of Stx1. Hope that manganese could be used as a treatment for STEC infection diminished, however, when Gaston et al. and additional work by Mukhopadhyay et al. showed that the protective effects of manganese did not extend to Stx2 [65,66]. Gaston and colleagues also showed that manganese was more toxic, both in cultured cells and in mice, than was reported by Mukhopadhyay and Linstedt. Our results show that manganese, unlike zinc, shows no protective effects on epithelial barrier function (measured as TER) or on Stx2 translocation across intestinal monolayers (Figure 3). Manganese did not inhibit ciprofloxacin-stimulated Stx2 production from STEC bacteria, unlike zinc (Figure 3A and B) and copper [12], and did not have any effect on *recA* expression (Figure 4F) or the SOS- induced bacterial elongation response (Additional file 1: Figure S1). Manganese has been shown to up-regulate expression of the Esps in STEC [67] and to increase basal Stx toxin production [12], so manganese has real potential to cause more harm than good in STEC infection. In addition, the neurotoxicity of manganese [68], which is worse in children and young animals, could exacerbate the Stx-induced encephalopathy that can accompany severe cases of STEC infection. Based on the literature mentioned and our results here, it appears that zinc is more likely to have therapeutic effects against STEC than manganese.

Copper also appears to have the ability to inhibit Stx production in an *recA*-independent fashion (Figure 4G and Ref. [12]), which is plausible given that *recA*-independent pathways are known to regulate Stx [69]. Copper, like zinc, also was able to block Stx2 translocation across intestinal monolayers (Figure 3F). Although copper is more toxic to humans than is zinc (based on the inverse ratios of the tolerable Upper Limits of these metals from the Food and Nutrition Board of the Institute of Medicine, available at https://fnic.nal.usda.gov/dietary-guidance/dietary-reference-intakes/dri-tables it is possible that copper might be combined with zinc to obtain additive effects via *recA*- dependent and *recA*-independent effects on STEC bacteria.

**Figure 7:**
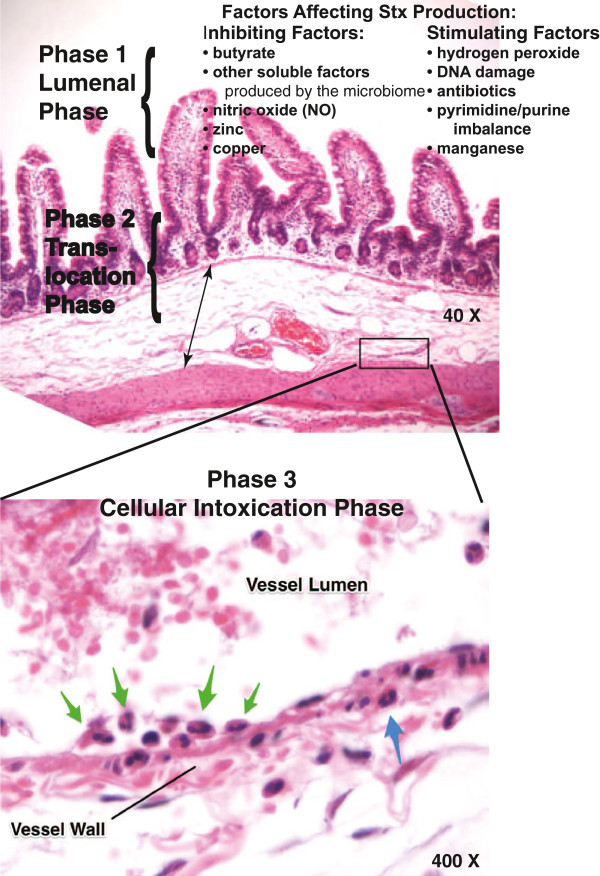
**Illustration showing multiple phases at which metals or other drugs might act to treat or prevent severe STEC infections.** Top panel, low power view of a rabbit ileal segment (“loop”) that had been treated with 3500 pg/mL Stx2 for 20 h, then fixed and stained with hematoxylin and eosin. The upper photograph demonstrates that Stx2 does not damage the enterocytes directly, as shown by the normal-appearing villi and crypts. The intestinal wall does show submucosal edema, however, a reproducible histological result of Stx exposure (double-headed arrow). Figure 7, lower panel, shows a higher power view of a blood vessel in the intestinal wall, showing abnormal adherence of polymorphonuclear leukocytes to the endothelial cells of the vessel wall (green arrows), as well as leukocytes in the vessel wall itself (blue arrow). Progression of similar vascular changes in vessels supplying the kidney and brain lead to the severe extra-intestinal sequelae of STEC infection, including hemolytic-uremic syndrome (HUS) and encephalopathy.

Mukhopadhyay and Linstedt focused their attention narrowly on the Gb_3_-expressing cells that are the target of Stx, while we believe that it may be more helpful to consider multiple steps in the natural history of STEC infection where interventions might help (Figure 7). Figure 7 and Additional file 2: Table S1 show that there are at least three separate phases at which zinc, other metals, or oral drugs might affect STEC after the pathogen enters the body. In the first phase, in the intestinal lumen, metals or other drugs might be able to prevent the expression of adhesins, virulence factors, and Stx (Figure 7, top portion). If the treatment was delayed, STEC infection was established, and Stx was produced, zinc or other interventions might still be able to reduce the amount of Stx which crosses the intestinal barrier (Figure 7, Phase 2). Previous literature on oxidant-mediated damage to intestinal epithelium has shown that tight junctions are the target of hydrogen peroxide [35,70,71] as well as the damage induced by nutrient deprivation [34,72]. Tight junctions are known to be regulated by extracellular divalent metals, especially calcium and zinc [34,73-77]. Based on previous research, therefore, we believe the effect of zinc on Stx translocation seen in Figures 1 and 2, and in Phase 2 of Figure 7, is likely due do its protective effect on the paracellular pathway rather than the transcellular/macropinocytosis pathway for Stx translocation that has also been well described [29,30].

Additional file 2: Table S1 summarizes the effects of zinc and four other metals in STEC and EPEC infection, based on results reported in this study as well as previous work by other investigators and our own laboratory. As can be seen from Additional file 2: Table S1, no other metal quite matches zinc in the wide number of different beneficial effects it exerts on host cells and inhibitory effects it exerts on the pathogen, although copper also shows some beneficial effects. In contrast, manganese, iron, and nickel all have the potential to worsen one or more aspects of STEC’s interactions with host cell (Additional file 2: Table S1).

EPEC adherence to host intestinal cells is heaviest in the ileum and cecum, and STEC adheres most strongly in the cecum and large intestine. Therefore, drugs or metals with limited absorption in the upper gastrointestinal tract would be ideal candidates for intervening at Phases 1 or 2 of Figure 7, because they would have to attain sufficient concentrations in the lumen of the distal gut; zinc salts fall into this category [12].

In the 3rd phase of Figure 7, Stx which has crossed the epithelial barrier binds to and begins to kill susceptible host cells, especially endothelial cells. Figure 7, lower portion, shows a higher power view of an intestinal blood vessel which has been affected by Stx2, showing adherence of polymorphonuclear leukocytes on the lumen of the endothelium (green arrows), as well as leukocytes which have been recruited into the wall of the vessel itself (blue arrow, showing a true vasculitis). When a similar process occurs in blood vessels elsewhere severe extra-intestinal complications can ensue. It appears that more research will be needed before we can declare we have drugs capable of blocking the 3^rd^ Phase of Stx action [14,65], and Additional file 2: Table S1.

Figure 7 illustrates possible points at which metals might act after STEC enters the intestinal tract of the host. Metals which prove too toxic to use in vivo in humans might still find use, however, in the “pre-ingestion” phase of STEC, i.e., in agricultural practices, during germination of sprouts, or during food processing to limit STEC adherence to fresh foods or block virulence. Indeed, copper has already attracted attention for its antimicrobial properties in this regard [78,79]. Divalent metals deserve additional research attention as inhibitors of bacterial virulence and enhancers of host defenses.

## Competing interests

The authors declare that they have no competing interests.

## Authors’ contributions

JB did the translocation experiments; RR developed the Miller assay and started the experiments with metals on *recA*; BW finished the experiments on *recA*, and tested metals on LEE4 and LEE5 expression. BW also measured bacterial elongation in response to SOS stimuli. SCB performed the bacteriophage plaque assays; JC planned experiments, compiled the data, and wrote drafts of the manuscript. All authors read and approved the final manuscript.

## Supplementary Material

Additional file 1: Figure S1 Ability of zinc to block the bacterial elongation (filamentation) response that ccompanies the SOS response. Panel **A**, Elongation response in STEC strain Popeye-1. Popeye-1 was subcultured at a dilution of 1:100 from an overnight culture in LB into DMEM medium and grown at 37° with 300 rpm shaking. After 1.5 h, ciprofloxacin was added to a final concentration of 4 ng/mL and incubation was continued for an additional 1.5 h. Bacteria were stained by mixing with an equal volume of 0.2% acridine orange in ethanol for 10 min, then the bacteria were washed twice by centrifugation (at 500 *g* for 10 min) and resuspension in 250 μl of water to remove excess acridine orange. The stained bacteria were spotted on glass microscope slides, allowed to dry, then examined by fluorescence microscopy under oil at 1000 X magnification. Panel **B**, effect of metals on ciprofloxacin-induced bacterial length in EPEC strain E2348/69. EPEC E2348/69 was grown in the absence or presence of 0.1 μg/mL ciprofloxacin ± various metals as shown. Bacteria were stained with acridine orange as described for Panel **A**, then photographed using a Retiga digital camera. Digital images were captured or converted to black-and-white, then subjected to image analysis using ImageJ, free image analysis software developed at the NIH. The version we used is called Fiji (ImageJ for MacIntosh, version 1.47n). Detailed instructions on how to open and process the files are available from the author at jcrane@buffalo.edu. Bacterial lengths were determined for each condition and expressed as a ratio compared to the no- ciprofloxacin, no-metal control bacteria. Panel **C**, effect of metals on bacterial elongation in STEC strain Popeye-1, using the same methods described for Panel **B**. Panel **D**, effect of zinc on mitomycin C-induced bacterial elongation. In Panel **D** the actual bacterial length is shown (in micrometers) using 2 micrometer size beads for calibration. Click here for file

Additional file 2: Table S1 Effects of Biometals at Multiple Phases of STEC and EPEC Pathogenesis. Click here for file
