# Supplementary material for: Zinc protects against shiga-toxigenic Escherichia coli by acting on host tissues as well as on bacteria
Source: BMC Microbiol. 2014 Jun 5;14:145. doi: 10.1186/1471-2180-14-145 (PMC4072484; doi:10.1186/1471-2180-14-145)
Supplement: Additional file 1: Figure S1 — Ability of zinc to block the bacterial elongation (filamentation) response that ccompanies the SOS response. Panel A, Elongation response in STEC strain Popeye-1. Popeye-1 was subcultured at a dilution of 1:100 from an overnight culture in LB into DMEM medium and grown at 37° with 300 rpm shaking. After 1.5 h, ciprofloxacin was added to a final concentration of 4 ng/mL and incubation was continued for an additional 1.5 h. Bacteria were stained by mixing with an equal volume of 0.2% acridine orange in ethanol for 10 min, then the bacteria were washed twice by centrifugation (at 500 g for 10 min) and resuspension in 250 μl of water to remove excess acridine orange. The stained bacteria were spotted on glass microscope slides, allowed to dry, then examined by fluorescence microscopy under oil at 1000 X magnification. Panel B, effect of metals on ciprofloxacin-induced bacterial length in EPEC strain E2348/69. EPEC E2348/69 was grown in the absence or presence of 0.1 μg/mL ciprofloxacin ± various metals as shown. Bacteria were stained with acridine orange as described for Panel A, then photographed using a Retiga digital camera. Digital images were captured or converted to black-and-white, then subjected to image analysis using ImageJ, free image analysis software developed at the NIH. The version we used is called Fiji (ImageJ for MacIntosh, version 1.47n). Detailed instructions on how to open and process the files are available from the author at jcrane@buffalo.edu. Bacterial lengths were determined for each condition and expressed as a ratio compared to the no- ciprofloxacin, no-metal control bacteria. Panel C, effect of metals on bacterial elongation in STEC strain Popeye-1, using the same methods described for Panel B. Panel D, effect of zinc on mitomycin C-induced bacterial elongation. In Panel D the actual bacterial length is shown (in micrometers) using 2 micrometer size beads for calibration. [file 1471-2180-14-145-S1.pdf]

Supplemental Fig. 1

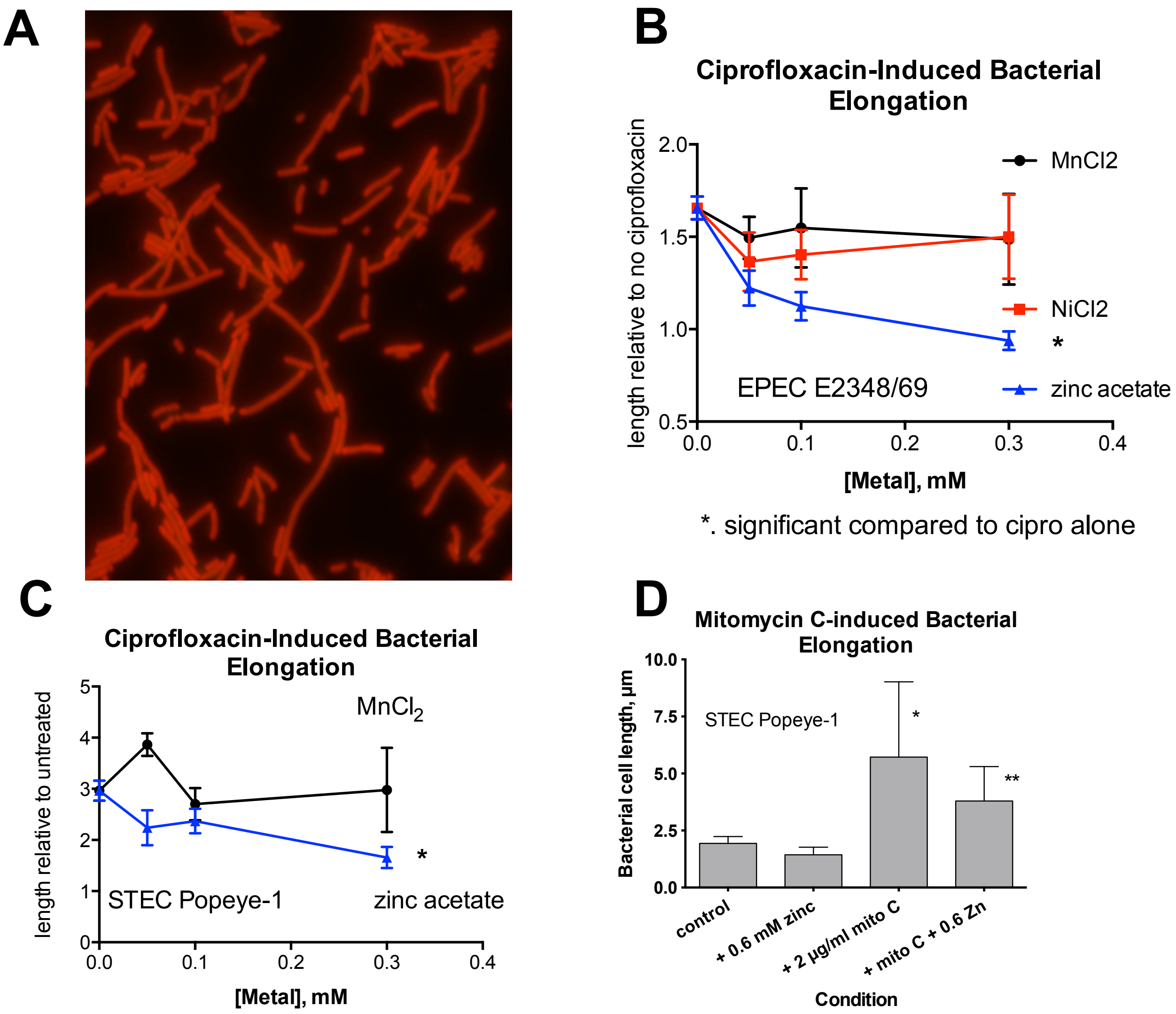

*. significant compared to cipro alone

*, significant compared to the no antibiotic control;
**, significant compared to mitomycin C treated

Legend for Supplemental Figure 1. Ability of zinc to block the bacterial elongation (filamentation) response that accompanies the SOS response.

Panel A, Elongation response in STEC strain Popeye-1. Popeye-1 was subcultured at a dilution of 1:100 from an overnight culture in LB into DMEM medium and grown at 37 ° with 300 rpm shaking. After 1.5 h, ciprofloxacin was added to a final concentration of 4 ng/mL and incubation was continued for an additional 1.5 h. Bacteria were stained by mixing with an equal volume of 0.2 % acridine orange in ethanol for 10 min, then the bacteria were washed twice by centrifugation (at 500 *g* for 10 min) and resuspension in 250 µl of water to remove excess acridine orange. The stained bacteria were spotted on glass microscope slides, allowed to dry, then examined by fluorescence microscopy under oil at 1000 X magnification.

Panel B, effect of metals on ciprofloxacin-induced bacterial length in EPEC strain E2348/69. EPEC E2348/69 was grown in the absence or presence of 0.1 µg/mL ciprofloxacin ± various metals as shown. Bacteria were stained with acridine orange as described for Panel A, then photographed using a Retiga digital camera. Digital images were captured or converted to black-and-white, then subjected to image analysis using ImageJ, free image analysis software developed at the NIH. The version we used is called Fiji (ImageJ for MacIntosh, version 1.47n). Detailed instructions on how to open and process the files are available from the author at jcrane@buffalo.edu. Bacterial lengths were determined for each condition and expressed as a ratio compared to the no- ciprofloxacin, no-metal control bacteria.

Panel C, effect of metals on bacterial elongation in STEC strain Popeye-1, using the same methods described for Panel B. Panel D, effect of zinc on mitomycin C-induced bacterial elongation. In Panel D the actual bacterial length is shown (in micrometers) using 2 micrometer size beads for calibration.
