# Supplementary material for: Zinc protects against shiga-toxigenic Escherichia coli by acting on host tissues as well as on bacteria
Source: BMC Microbiol. 2014 Jun 5;14:145. doi: 10.1186/1471-2180-14-145 (PMC4072484; doi:10.1186/1471-2180-14-145)
Supplement: Additional file 2: Table S1 — Effects of Biometals at Multiple Phases of STEC and EPEC Pathogenesis. [file 1471-2180-14-145-S2.pdf]

Supplemental Table 1. Effects of Biometals at Multiple Phases of STEC and EPEC Pathogenesis.

| | Lumenal Phase | | | | | Epithelial Barrier and Translocation Phase | | Stx Cellular Intoxication Phase | Reference(s) |
|---|---|---|---|---|---|---|---|---|---|
| Metal: | LEE and adhesins | Cipro-induced *recA* | Cipro-induced *stx* RNA | Basal Stx Toxin protein | Cipro-induced Stx Toxin protein | Effects on $H_2O_2$-induced Epithelial Damage | Translocation of Stx Across Epithelium | Ability to Block Cell Death | |
| Zinc | ↓ | ↓↓ | ↓↓ | ↓↓ | ↓↓ | ↓ | ↓↓ | n.d. | This study |
| Copper | n.d. | --- | ↓ | n.d. | ↓↓ | --- | ↓↓ | n.d. | This study and [1] |
| Manganese | ↑ | --- | ↓ | ↑ | --- | --- | ---; ↑ at 0.5 mM | ↓ for Stx1 in Hela cells; no effect on Stx2 | [1-4] and this study |
| Iron | n.d. | --- | ↑ | n.d. | n.d. | --- or slight ↑ | --- or slight ↑ | n.d. | [1] |
| Nickel | n.d. | --- | ↓↓ | ↑ | n.d. | --- | --- | n.d. | [1] |

Legend: ---, no effect; ↓↓, ≥ 75% reduction; ↓, 50 to 75 % reduction; ↑, ≥ 2-fold increase; n.d., not done

References Cited in Supplemental Table


1. Crane JK, Byrd IW, Boedeker EC: **Virulence Inhibition by Zinc in Shiga-Toxigenic Escherichia coli.** *Infection and immunity* 2011, **79:**1696.
2. Beltrametti F, Kresse AU, Guzmán CA: **Transcriptional Regulation of the esp Genes of Enterohemorrhagic Escherichia coli.** *Journal of Bacteriology* 1999, **181:**3409-3418.
3. Mukhopadhyay S, Linstedt AD: **Manganese Blocks Intracellular Trafficking of Shiga Toxin and Protects Against Shiga Toxicosis.** *Science* 2012, **335:**332-335.
4. Gaston MA, Pellino CA, Weiss AA: **Failure of Manganese to Protect from Shiga Toxin.** *PLoS ONE* 2013, **8:**e69823.
